# Structural and functional effects of mechanical ventilation and aging on single rat diaphragm muscle fibers

**DOI:** 10.1186/cc14330

**Published:** 2015-03-16

**Authors:** N Cacciani, H Ogilvie, L Larsson

**Affiliations:** 1 Karolinska Institutet, Solna - Stockholm, Sweden

## Introduction

The still unclear mechanisms causing ventilator-induced diaphragm dysfunction (VIDD) are considered intrinsic to the diaphragm muscle fibers. VIDD delays and complicates weaning from mechanical ventilation (MV) and accordingly contributes to prolonged ICU stay by 50%, with older patients being more affected than the young. The main aim of this study was to measure the effects of aging and 5 days of MV on rat diaphragm muscle fiber structure and function. We also aimed to investigate the biological age of the old rats to obtain data useful to design future experimental studies focusing on the effects of age in an ICU setting.

## Methods

We used a unique ICU rat model, which allows us to maintain the vital parameters stable under deep sedation and MV for long durations (several weeks). Diaphragm fiber cross-sectional area (CSA) and force-generating capacity (specific force = absolute force / CSA) were measured in young (6 months) and old (28 to 32 months) F344/ BN hybrid rats in response to 5 days of deep sedation and volume-controlled MV. To investigate the biological age of the old rats, we performed a second set of experiments, comparing muscle fiber CSA and specific force in fast and slow-twitch distal hind limb muscles in three different age groups: young adults (6 months), middle aged (18 months) and old rats (28 months).

## Results

This study demonstrated an unexpected increase in CSA (*P *< 0.001) of the diaphragm fibers in response to 5 days of MV in both young and old animals. Maximum force decreased 39.8 to 45.2% (*P *< 0.001) in both young and old animals compared with controls, resulting in a dramatic loss of specific force. This increase in CSA and the concomitant decrease in specific force observed in both young and old diaphragm fibers are compatible with an ineffective compensatory hypertrophy in response to the MV. The comparison of the limb muscles fibers from young, middle aged and old animals confirmed the 28 to 32 month rats to be senescent from a skeletal muscle point of view.

## Conclusion

These results demonstrate intrinsic changes in diaphragm muscle fibers of significant importance for the prolonged and complicated weaning from MV. Moreover, the increased number of frail diaphragm muscle fibers observed after MV in old age, both controls and mechanically ventilated, offers a further age-related possible mechanism which may be of significant clinical importance. These results also provided useful information to design future experimental studies focused on the effect of age in an ICU setting, pharmacological intervention strategies as well as mechanisms underlying rat strain differences.

